# Autoimmunity: the neoantigen hypothesis

**DOI:** 10.3389/fimmu.2024.1432985

**Published:** 2024-06-27

**Authors:** Tomas Mustelin, Felipe Andrade

**Affiliations:** ^1^ Division of Rheumatology, Department of Medicine, University of Washington, Seattle, WA, United States; ^2^ Division of Rheumatology, The Johns Hopkins University School of Medicine, Baltimore, MD, United States

**Keywords:** neoantigen, autoimmunity, autoantibodies, citrullination, rheumatoid arthritis, systemic lupus erythematosus, transposable elements

## Cracks in the ‘loss of tolerance’ concept

Chapters and text books about autoimmunity typically begin with the statement that autoimmunity arises as a consequence of the ‘loss of tolerance’, which is described as intrinsic to T cells (1) and/or B cells (2), which, therefore, begin to react against self-antigens. On the surface this seems perfectly logical: the difference between a healthy young person with full immunological tolerance to ‘self’ and the same individual a decade later diagnosed with an autoimmune disease, is, clearly, that the latter has a devastating reactivity of their immune system against ‘self’. The unspoken assumption here is that ‘self’ in the diagnosed patient is identical to ‘self’ in the same individual at earlier time points in their life before symptoms developed. This is the first of four problem areas with the ‘loss of tolerance’ concept that we will discuss.

### Dilemma 1 – the unchanging self?

Rheumatoid arthritis (RA) represents a well-known exception to the notion that all self-derived peptides that T cells develop central and peripheral tolerance for during childhood and adolescence remain the same throughout life. In this disease, a substantial portion of self-reactive T cells recognize epitopes in which arginine residues have been post-translationally modified into citrulline by deimination (3–6). In concert with the activated T cells, B cells generate autoantibodies against numerous citrullinated proteins to the extent that these antibodies have become diagnostic of the disease (7). Is this ‘loss of tolerance’? In our stricter molecular definition of the concept, it is not. Rather, it is the response to antigens never seen by the immune system before. Physiological citrullination is limited and does not provoke an immune response, while the much more extensive citrullination in RA of many proteins (8, 9) that are not normally citrullinated creates ‘neo-autoantigens’ that are indistinguishable from pathogen-derived antigens (10–12). Hence, the immune system will do exactly what it is supposed to: activate and make every effort to eliminate the danger.

Numerous other examples of post-translationally generated neoantigens in autoimmune diseases have accumulated in recent years (13–15). In type 1 diabetes (T1D), autoantigens include citrullinated β-cell antigens (16–18), insulin peptides covalently cross-linked to other peptides by transglutaminase (17), and peptides from abnormally spliced transcripts (19). This is also true in multiple sclerosis, SLE, scleroderma, Sjögren’s syndrome, and autoimmune myositis (15, 20).

### Dilemma 2 – features of clinical disease, autoantibody repertoires, and responses to therapy

The second shortcoming of the loss of tolerance dogma is its inability to explain many of the basic aspects of autoimmune diseases as we observe them in human patients. If autoimmunity was initiated by a molecular mechanism causing T cell-intrinsic loss of tolerance, the antigens seen by these T cells would likely be stochastic, resulting in a broad spectrum of autoimmune syndromes against a myriad of autoantigens. This is not what we see in the clinic. Rather, we observe a modest number of distinct disease entities, each with a characteristic prevalence, gender ratio, peak age of onset, clinical course and prognosis, a set of unique autoantibodies, a spectrum of typical symptoms and organ manifestations, and response to different medications. Clinically, autoimmune diseases can be remarkably different from each other, and they are managed by different specialists: rheumatologists, gastroenterologists, endocrinologists, neurologists etc. It is difficult to believe that all these diseases would be initiated by the same type of molecular mechanism, *i.e.*, loss of tolerance in T and/or B cells.

Although currently used drugs are generally immunosuppressive (largely because of the reliance on mouse models for their discovery and development (21)), it is remarkable how differently autoimmune diseases respond to specific medications. For example, psoriasis improves dramatically after interleukin 17 blockade (22, 23), while RA does not (24, 25). Instead, most RA patients respond well to TNF blockers (26, 27), while patients with systemic lupus erythematosus (SLE) do not (28, 29). B cell depletion by rituximab works well in RA (30, 31) and multiple sclerosis (32), but not in SLE (33). Interestingly, however, these therapeutic effects match our understanding of different flavors of immune responses to different classes of antigens: bacterial, viral, carbohydrates, and nucleic acids. On the other hand, the loss-of-tolerance hypothesis struggles to provide an explanation for what becomes an autoantigen. Evidence is lacking for ideas like antigen overexpression (e.g. in tumors), molecular mimicry, autoimmune ‘pre-disease’, or medication. Mass action does not apply to the immune system, which seeks low-abundance antigen and is tolerized or anergized by higher amounts of antigen. Molecular mimicry, which postulates that a foreign antigen that is sufficiently similar to a self-antigen can trigger an immune response against this self-antigen, may explain tendencies towards autoimmunity during severe infection, e.g. by SARS-CoV2, but tends to fade as soon as the exogenous antigen is cleared. To the best of our knowledge there are no cases of proven molecular mimicry driving chronic autoimmune disease. The concept of pre-autoimmune disease is interesting and in line with what we propose as the neoantigen hypothesis.

There is also a wealth of evidence in support of environmental factors (34–36), such as infectious agents, the gut microbiome, cigarette smoking, and ultraviolet light exposure, influencing the pathogenesis and/or triggering exacerbations in human autoimmune conditions. It is not obvious how one would fit all of these into a model where T cells have lost the tolerance for self-antigens.

### Dilemma 3 – genome-wide association studies

The genetic underpinnings of autoimmune disorders have been explored over the last two decades, particularly through GWAS (37), which rely on common single-nucleotide polymorphisms, *i.e*., the minor allele is present in sufficient frequencies in the general population. An allele is deemed disease-associated if its frequency is higher in the disease than in the healthy control group. Importantly, the sufficiently high frequency of the disease-associated allele in the population means that it has been positively selected for over evolutionary time, presumably because it conferred a survival advantage to the carrier. Known examples of this include the role of the autoimmune-associated allele of *PTPN22 (* 38, 39) in resistance to viral infection (40) and tuberculosis (41). Apparently, the immune system in carriers of the disease-associated PTPN22*W620 allele respond more appropriately to certain foreign antigens. Similarly, autoimmune-associated HLA haplotypes, such as HLA-DR4/DQ8, DR2/DQ6 and DR3/DQ2, may have been selected for their ability to present microbial peptides and promote the production of anti-microbial cytokines (42). Consequently, the disease-associated alleles do not tell us that the immune system has gone awry or that autoimmunity starts by loss of tolerance. They do, however, support the notion that the sensitivity of the immune system is an important factor.

Another category of genes associated with autoimmune disease are those that are not clearly immune-related. For example, several genes associated with autoimmune diseases, particularly SLE, are involved in the clearance of DNA (43). Another example is the association of RA with the genes *PADI2* (44, 45) and *PADI4* (46), which encode the two citrullinating enzymes that participate, alone or in combination, in the production of citrullinated neoantigen in this disease.

### Dilemma 4 – recent findings through deep immune profiling

Recent advances in ultra-sensitive DNA sequencing technology now allow researchers to profile immune cells at the single-cell level for transcriptional repertoire and whole-genome epigenetic landscapes. Publications built on this new capability are now frequent in top journals and they promise new drug targets and the identification of the pathogenic cell. Unfortunately, nearly all these studies compared immune cells between disease tissue and healthy tissue, one disease at a time. While skewing of immune cell lineages are observed, and large sets of genes are expressed differently, the main take-home message is that the immune system is activated. As these studies were conducted in more and more diseases, it became apparent that the findings were quite similar in each disease. Particularly striking was the observation that the immune response in COVID-19 patients is very similar to that in autoimmune disease. Despite the differences between an exogenous virus like SARS-CoV2 and the unknown causes of RA or SLE, the immune response is very similar. We reported that changes in gene expression is near-identical in neutrophils from COVID-19 and SLE; only one single gene was statistically significantly regulated in opposite directions (47). No smoking gun. All the immune cells observed in autoimmune conditions represent lineages and phenotypes present in healthy individuals, particularly during viral or bacterial infections. Despite the frequent use of the term ‘dysregulated’, it seems to us that all the observations reflect a normal (‘eu-regulated’) immune activation with precious few, if any, disease-specific abnormalities that would illuminate the cause or pathogenesis of the disease in question. This is precisely what you would expect to see if autoimmune disease was the result of neoantigens.

## The neoantigen hypothesis

Given the above shortcomings of the loss of tolerance concept, we propose another model for the initiation and progression of autoimmune disorders. We posit that it is not a malfunction of the immune system that leads to autoimmunity, but that aberrantly generated neoantigens are the cause of an immune response that may be asymptomatic at first, but then gradually escalates over a longer period of time into a clinically manifest condition that can be given a specific diagnosis. By ‘aberrantly generated’ we mean that these neoantigens are not present during normal development, so tolerance for them cannot develop, but that they are produced later in life by molecular mechanisms unique to a specific autoimmune disease in individuals who subsequently develop that disease. These currently poorly understood molecular mechanisms can be considered the upstream and real cause of the disease, while the immune system that reacts to the neoantigen is executing its preprogrammed function, even if that is driving the inflammation that underlies the symptoms and organ manifestations of the disease. The immune system is responding exactly as it would against any ‘foreign’ antigen and it will stay activated until the antigen is eliminated. The problem is that the source of the neoantigen is not under the immune system’s control.

Unlike the loss of tolerance concept, the neoantigen hypothesis postulates that ‘self’ indeed can change. It also fits the basic immunological concept that the nature of an immune response depends on the type of antigen. The molecular mechanism that generates a particular kind of neoantigen determines when and where that occurs. It may be restricted to a cell type or organ, such as the salivary gland in Sjögren’s syndrome, or skeletal muscle in myositis, or it may be widespread. Neoantigen production could be episodic, resulting a waxing and waning immune response, or chronic and stable. It may start at a certain age, it may have a sex bias, and it may be influenced by the risk factors of autoimmunity, such as smoking. Carriers of disease-associated alleles – like PTPN22*W620, DR2 (DRβ1*1501)/DQ6 (DQβ1*0602), DR3 (DRβ1*0301)/DQ2 (DQβ1*0201), and DR4 (DRβ1*0401)/DQ8 (DQβ1*0302) – may respond more robustly to neoantigens. Non-immune genes with disease-associated alleles may have a role in the generation of neoantigens. Monogenic SLE caused by *DNASE1L3* deficiency (42, 48) is an excellent example of how a non-immune gene involved in the clearance of an antigen (*i.e.*, DNA) has a dominant effect in the development of an autoimmune disease in the context of a normal immune system. The neoantigen hypothesis also predicts that an immune response to neoantigen should look like a normal immune response to exogenous antigens, just as we observe in the deep immune profiling of patient immune cells and tissues.

Whether the neoantigens are present episodically or in a more continuous manner, the immune system may become frustrated by its persistence and escalate to levels of activity that include tissue damage and epitope spreading. The latter may broaden the set of involved antigens and may encompass *bona fide* autoantigens, as occurs during more severe and longer lasting COVID-19. However, we speculate that, just as in COVID-19, this broader reactivity would subside over some period of time if the neoantigens vanish.

## Sources and molecular mechanisms of neoantigen production

**Figure 1:**
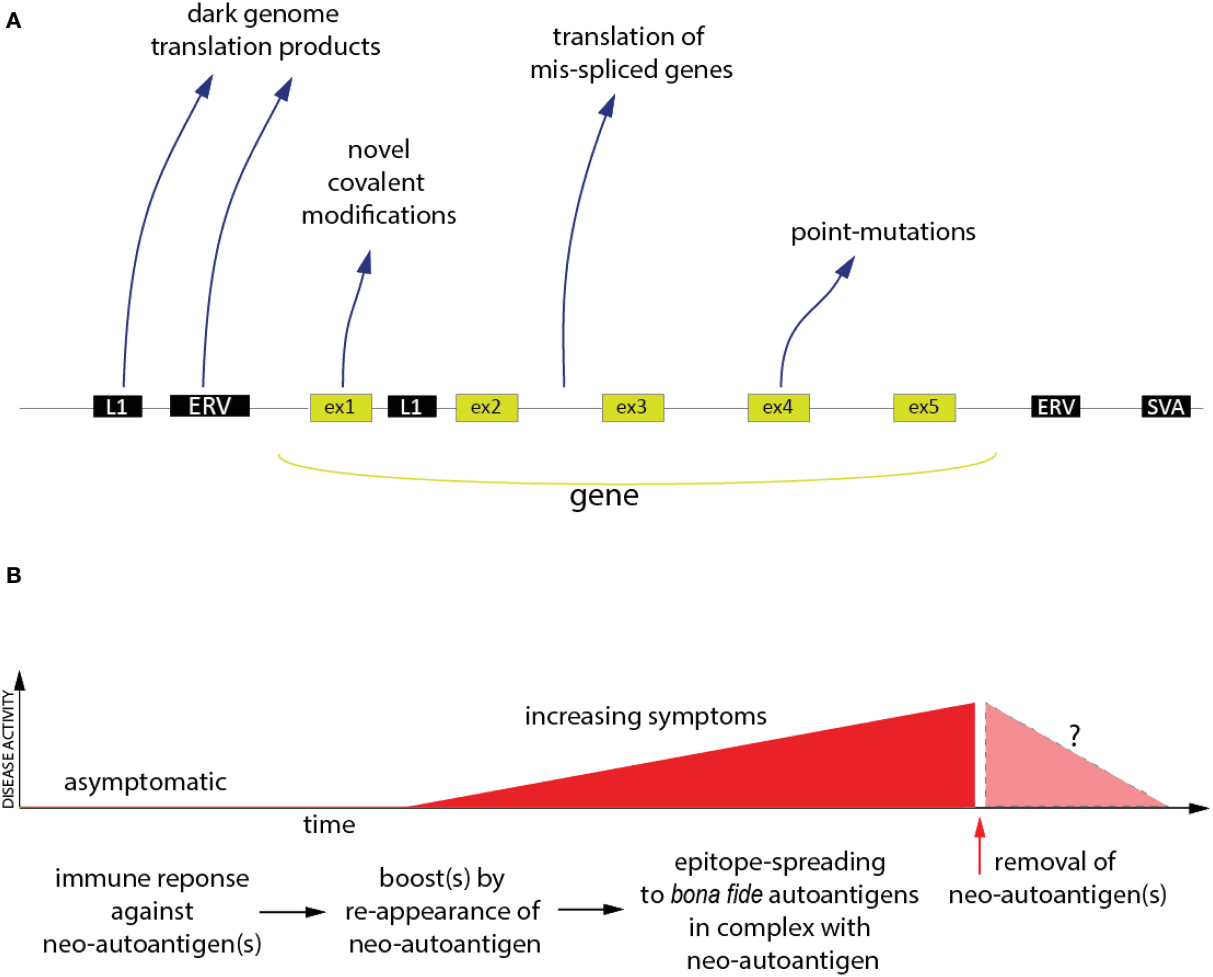
The Neoantigen Hypothesis. **(A)** The genomic and post-translational sources of the four principal categories of neo(auto)antigens. Novel covalent modifications are not restricted to exon-encoded proteins, but could, in principle, also affect the other classes of neoantigen polypeptides. **(B)** Proposed time-course of events in the pathogenesis of an autoimmune disease (red) driven by neo(auto)antigens and the hypothetical consequence of removal of the neoantigens (pink).

Research of anti-tumor immunity was greatly stimulated by the introduction of checkpoint inhibitors, such as the antibodies to CTLA-4 (ipilimumab) and PD-1 (pembrolizumab), over a decade ago. Anti-tumor immunity is a form of autoimmunity, in which the immune system is recognizing and responding to neoantigens derived from altered ‘self’. These antigens are now well-accepted in the immuno-oncology community and generally fall into four distinct categories, schematically shown in **Figure 1**: (i) epitopes generated by point-mutations or other genetic changes in the malignant cells; (ii) the translation of transcripts resulting from aberrant mRNA splicing (49, 50); (iii) peptides and proteins encoded by retroelements and endogenous retroviruses (51–53), many of which are expressed at elevated levels in malignant cells; (iv) aberrant post-translational modifications.

While these antigens are instrumental for the success of cancer immunotherapy, a causative role of similar neoantigens in non-cancer autoimmunity remains speculative. In fact, they are generally ignored by the autoimmunity research community. Nevertheless, there are recent publications showing that all four categories of tumor neoantigens are also present in patients with non-cancer autoimmune disorders. There is also a tantalizing co-occurrence of certain rheumatic disease with tumors, both characterized by shared autoantibodies (54).

Point-mutations have been reported in Vacuoles, E1 enzyme, X-linked, Autoinflammatory, Somatic (VEXAS) syndrome, in clonal hematopoiesis (55), and in RA (56–58). So far, these studies have focused on point-mutations altering the function of genes, not as neoepitopes. There are several reports of dysregulated mRNA splicing in SLE (59, 60), which can lead to translation products predicted (61) to contain stretches of novel amino acids due to frame shifts or retained parts of introns. We have shown that mRNA splicing is particularly aberrant in SLE neutrophils (Najjar et al., 2024)^1^, in which 80 events resulted in novel amino acid sequences. Every patient in our study (n=15) had at least one such event, while some had many, one as many as 36 of them (Najjar et al., 2024)^1^. In another example, a subset of autoantibodies against Ro52 were found to be directed towards a unique domain due to a frameshift in a splice variant found in SLE neutrophils (62). Proteins and peptides from non-exonic regions of our genome are present in increased quantities in several rheumatic diseases (61, 63, 64) and autoantibodies against them (65–68) are prevalent.

The best example of neoantigens created by post-translational modification is in RA (3), but there are many other examples of post-translational modifications creating neoantigens in autoimmune diseases, such as carbamylation (69), acetylation, cysteine carboxyethylation (70) and covalent metabolite attachment (71). As is well-documented, self-proteins covalently modified by a small-molecule drug, termed a hapten, can be very immunogenic and drive a dangerous immune response (72).

Post-translational neoantigens can also be generated in the context of infection through the interaction of viral and host products, as well as during the killing of infected cells by cytotoxic cells. For example, the autoantigen La binds to virus-associated (VA) RNA in adenovirus-infected cells (73), as well as EBV-encoded RNAs (EBER1 and EBER2) during EBV infection (74). Moreover, numerous autoantigens are modified as result of proteolysis in target cells killed by cytotoxic cells (15). Interestingly, the autoantigen Ro60 is in complex with endogenous Alu retroelements and anti-Ro60-Alu RNA immune complexes are found in circulation in SLE (75).

## Drug development

The neoantigen hypothesis predicts that immunosuppressive drugs will reduce disease activity in autoimmune conditions. This is largely the case but comes at the cost of reducing immune responses against exogenous antigens to a similar extent. The neoantigen hypothesis also opens a completely new avenue for drug development, namely the opportunity to stop the process responsible for neoantigen production. Since this process likely is active only in patients with the disease, blocking it should be very safe. At present, we have an incomplete understanding of the mechanisms that generate neoantigens, and most of what we know is in the context of tumors, where the desire is to increase them for the benefit of immunotherapy. In non-cancer autoimmunity, the goal will be to reduce or eliminate these processes.

## Concluding remarks and future perspectives

The neoantigen hypothesis seeks to stimulate a critical rethinking and re-examination of the current loss of tolerance concept. We do not wish to state that the loss of tolerance concept is wrong, but we believe it is obvious that other mechanisms must also be at play. Defining the primary role of neoantigens vs. loss of tolerance in autoimmune diseases has critical implications for pathogenesis, prevention and treatment. Progress in understanding the molecular pathways that generate neoantigens will enable the design of new preventive and therapeutic approaches, which could be applied prior to the preclinical detection of autoantibodies or once the disease has been established without interfering with the normal function of the immune system. Eventually, this approach will demonstrate in clinical trials whether neoantigens are important in precipitating or perpetuating autoimmune conditions and their exacerbations.

## Author contributions

TM: Conceptualization, Data curation, Formal analysis, Funding acquisition, Investigation, Methodology, Project administration, Resources, Software, Supervision, Validation, Visualization, Writing – original draft, Writing – review & editing. FA: Conceptualization, Formal analysis, Funding acquisition, Methodology, Writing – original draft, Writing – review & editing.

## Footnotes

1 Najjar R, Wang X, Pineda JMB, Alessi H, Bays A, Bradley RK, et al. Altered protein structures and neoepitopes in lupus neutrophils from dysregulated splicing of messenger RNA. ACR Open Rheumatol. (2024). in press.
